# Identifying central dimensions of quality of life including life-related values, preferences and functional health in older patients with cancer: a scoping review protocol

**DOI:** 10.3389/fpsyg.2024.1455825

**Published:** 2024-10-22

**Authors:** Franziska Springer, Ayumu Matsuoka, Kyoko Obama, Anja Mehnert-Theuerkauf, Yosuke Uchitomi, Maiko Fujimori

**Affiliations:** ^1^Department of Medical Psychology and Medical Sociology, Comprehensive Cancer Center Central Germany (CCCG), University Medical Center Leipzig, Leipzig, Germany; ^2^Division of Supportive Care, Survivorship, and Translational Research, National Cancer Center, Institute for Cancer Control, Tokyo, Japan; ^3^Department of Cancer Survivorship and Digital Medicine, The Jikei University School of Medicine, Tokyo, Japan

**Keywords:** cancer, elderly cancer patients, quality of life, geriatric, scoping review

## Abstract

**Background:**

Older patients with cancer already represent the largest proportion of cancer survivors which will further increase in the upcoming years. However, older patients are highly underrepresented in clinical research, leading to a detrimental knowledge gap. Research on important aspects of quality of life (QoL) and associated factors for older patients with cancer is insufficient to date.

**Aim:**

The objective of this scoping review therefore is to investigate the dimensions of QoL including functional health, life-relevant values and preferences in older adults with cancer across all tumor entities and health care settings. It will further identify medical, sociodemographic, psychosocial and geriatric aspects associated with QoL in the elderly and compare these with younger cancer patients and older non-cancer cases.

**Methods:**

Published articles investigating QoL dimensions and associated factors in older patients with cancer, i.e., exclusively patients ≥65 years or mean/median age ≥ 70 years for age-mixed samples, or that compare results of older with younger cancer patients or with older non-cancer cases will be considered for this scoping review. Older patients with cancer across all tumor entities, disease stages and health care setting will be included. PubMed and PsychINFO databases will be searched for relevant articles. Abstracts and titles will be screened for basic inclusion, and two independent reviewers will conduct a full text screening to evaluate the age criteria and decide on the final inclusion of the study. Data on study and participant characteristics, QoL dimensions and geriatric factors will be extracted using a data extraction sheet. Results will be summarized descriptively to address the objectives of this review.

**Discussion:**

The findings of this scoping review will provide valuable insights into central dimensions of QoL, including values, preferences and functional health in older adults with cancer, and help to improve targeted interventions and healthcare planning.

## Introduction

The burden of cancer and its role as a leading cause of death worldwide is increasing. In 2020, 19.3 million new cancer cases and almost 10 million cancer-related deaths occurred worldwide (Sung et al., 2021). Cancer patients nowadays may survive longer after their cancer diagnosis, which reflects our aging society, as well as improvements in cancer diagnostics and treatment. As the number of cancer survivors is increasing constantly, patients grow older while living with a cancer disease and its consequences. In addition, there are higher incident rates among elderly patients. Currently, older adults with cancer already represent the largest proportion of cancer patients (Pilleron et al., 2022; Pilleron et al., 2021; Xiang et al., 2022), especially in Europe, eastern Asia and North-America (Pilleron et al., 2021; Xiang et al., 2022), with more than two thirds of new cancer cases being patients above the age of 60 years (Xiang et al., 2022).

Most studies define the term “elderly” as patients above the age of 65 years, whereas other studies include patients older than 60 years. A common classification therefore differentiates between “young-old” (65–74 years), “middle-old” (75–84 years) and “old-old” (≥ 85 years) (Shenoy and Harugeri, 2015). The number and proportion of older patients with cancer will further increase in the upcoming years, which poses a growing challenge to survivorship care (Atun and Cavalli, 2018), especially in terms of addressing the specific needs of the elderly.

Managing cancer in elderly patients is a complex challenge (Williams et al., 2016; Higuera et al., 2016; Bellury et al., 2011). The population of older patients with cancer is very heterogeneous in terms of intrinsic health capacities, including mental health and cognitive capacities, comorbidities, impairments and frailty, as well as in terms of performance activities such as social interactions, work and mobility (Bickenbach et al., 2023). As a result, their functional health and health care needs vary significantly.

Supportive care needs in older patients may differ substantially from those of younger cancer survivors. Unmet supportive care needs have been shown to negatively impact quality of life (QoL) in cancer patients (Hansen et al., 2013; Cochrane et al., 2022). The aim of supportive cancer care therefore is to support patients holistically with their individual needs and to improve their QoL (Epstein and Street, 2007). Health-related QoL is understood to be multifaceted, combining physical, emotional, cognitive and social aspects (Hays and Reeve, 2008). The assessment and evaluation of QoL in cancer patients thus combines the patients’ global health, functionality and physical symptoms (Fayers and Bottomley, 2002).

So far, the evidence regarding QoL in older adults with cancer is contradictory. Some studies have shown that older patients adapt well to the cancer disease and are less affected by mental and social health problems, resulting in an overall better global QoL (Arraras et al., 2018; Verweij et al., 2018; Thong et al., 2019). Other studies report worse QoL across various dimensions (Babcock et al., 2020; Mamguem Kamga et al., 2021). QoL assessment tools used in clinical care are well validated in the oncological setting, the most commonly used tool being the QoL questionnaire by the *European Organization for Research and Treatment of Cancer* (EORTC) (Fayers and Bottomley, 2002). Such tools, however, were mostly developed for the entire population of cancer survivors and might therefore lack sensitivity to important dimensions and values for older patients as well as reference to age-specific areas of burden (Fitzsimmons et al., 2009).

Older patients with cancer are an underrepresented population in clinical research (Hurria et al., 2015; Talarico et al., 2004). Despite extensive research and systematic reviews on QoL in diverse oncological populations and treatment settings (Van Leeuwen et al., 2018; Mokhtari-Hessari and Montazeri, 2020), clinical trials are rarely tailored to the elderly, leading to a lack of knowledge regarding treatments, important needs, and clinical endpoints. Clinicians must rely on knowledge from younger and healthier populations that may not be directly applicable to older patients. This is particularly relevant for important aspects of health-related QoL in oncological populations. Thus, despite the increasing number of older patients with cancer, there is still relatively little knowledge about their QoL, functional health, values, and preferences regarding important aspects of care. It is also not well understood to what extent sociodemographic and medical factors common among older patients, such as widowhood and small social networks, socioeconomic constraints, physical comorbidities, and geriatric aspects have a significant impact on different dimensions of QoL, including physical or emotional functioning. Bellury and colleagues conclude in their integrative review about elderly cancer survivorship that a robust knowledge base for older cancer survivors is needed to improve treatment and intervention tailoring, survivorship independence and to prevent a decline into frailty (Bellury et al., 2011).

A preliminary search for reviews was conducted to identify the latest reviews regarding important aspects and dimensions of QoL in older patients with cancer. However, no current or ongoing systematic or scoping reviews on the topic were identified. The few existing systematic and scoping reviews are either not up-to-date (Fitzsimmons et al., 2009; Wedding et al., 2007), or targeted to specific populations, such as those with solid tumors undergoing adjuvant chemotherapy and/or radiotherapy (Cheng et al., 2018).

To inform survivorship care planning of older patients with cancer, it would be valuable to better understand QoL, relevant dimensions and potential knowledge gaps. The objective of this scoping review therefore is to investigate relevant dimensions of QoL, including life-related values, preferences, and functional health in older patients with cancer, as well as to identify important medical, sociodemographic, psychosocial and geriatric factors associated with QoL.

### Objective

This scoping review aims to identify central dimensions of QoL in older patients with cancer reveal important QoL-related values, preferences and functional health aspects determine the medical, sociodemographic, psychosocial and geriatric factors associated with QoL in older patients with cancer, including comparisons with younger cancer patients and with older non-cancer cases.

## Methods

This scoping review will be conducted according to the JBI methodology for scoping reviews (Peters et al., 2020) and the Preferred Reporting Items for Systematic reviews and Meta-Analyses extension for Scoping Reviews guidelines (PRISMA-ScR) (Tricco et al., 2018).

### Inclusion criteria

Table 1 summarized the procedure of this scoping review. We will consider articles that include populations of older patients with cancer of all tumor entities (International Classification of Diseases, 10^th^ edition (ICD-10): C00-C96). We will consider articles that either address exclusively older cancer survivors, i.e., ≥ 65 years, or, if an age-mixed sample is investigated, patient samples with a mean/median age ≥ 70 years, to ensure that the majority of the sample consist of older cancer survivors. Age range and percentage of older cancer patients for age-mixed studies will be reported.

**Table 1:** Summary of scoping review procedure.

Study population	Cancer patients of all tumor entities were included, focusing either exclusively on older cancer survivors (≥ 65 years), or, for age-mixed samples, with a mean/median age ≥ 70 years
Concept	Studies reporting on (1) outcomes of validated QoL assessment tools (global QoL and dimensions), (2) comparing QoL dimensions of younger and older cancer patients, or (3) comparing QoL dimensions of older cancer patients and older non-cancer controls
Context	All countries, regions, and health care settings
Types of sources	
Inclusion	Published articles in English language presenting quantitative, qualitative or mixed methods studies
Exclusion	Efficacy/Effectiveness trials using QoL as outcome, reviews, meta-analyses, case studies, case series, opinion pieces, editorials, study protocols, conference papers
Study selection	Check for basic inclusion criteria (e.g., no efficacy trial, review); Check for age criteria (see study population); Full text screening (e.g., exclude if no QoL results reported)
Data extraction	Study characteristics, patient characteristics, QoL outcomes, associated factors
Data analysis	Descriptive statistics on included studies and patient characteristics. QoL results will be summarized descriptively and associated factors will be categorized to identify overarching topics.

Cancer patients will be included across all types of health care settings, i.e., inpatient, outpatient, curative and palliative care, as well as different treatment stages, i.e., newly diagnosed cancer patients, during and after cancer treatment and long-term survivors. This will ensure a comprehensive investigation of important aspects of QoL and functional health for older cancer patients.

The concept of this review will consider studies investigating and/or reporting (i) outcomes of validated QoL assessment tools (global QoL and dimensions of QoL) including functional health, values and preferences in a population of older patients with cancer, (ii) comparing QoL dimensions of younger and older cancer patients, or (iii) comparing QoL dimensions in older cancer patients with older non-cancer controls. If a study includes different age groups, it will only be included, if results are reported separately for the elderly subsample. In addition, studies that investigate and report sociodemographic, medical and psychosocial factors associated with QoL as well as geriatric assessments associated with QoL will be considered for this scoping review.

The context will be studies of all countries, regions and health care settings investigating QoL in older patients with cancer.

As types of sources, this scoping review will consider published articles that present quantitative, qualitative, and mixed methods study designs. Efficacy or effectiveness trials using QoL outcomes as endpoints, reviews (e.g., systematic, scoping, narrative), meta-analyses, case studies, case series, opinion pieces, editorials, study protocols and conference papers will be excluded.

### Search strategy

A comprehensive literature search using the databases PubMed and PsychINFO is conducted in February 2024. Articles will be included that were published in English until January 2024. Based on a previous study investigating QoL dimensions across disease-free cancer survivors, in order to develop a questionnaire that captures the full range of QoL dimensions (Van Leeuwen et al., 2018), the following search terms will be used: (“Survivors”[Major] OR “Survivors/psychology”[Major]) AND (“neoplasms”[Major] OR “Carcinoma”[Major]) AND (“Quality of Life”[Mesh] OR “patient-reported outcomes” OR “health-related quality of life” OR “wellbeing” OR “well-being” OR “Mental Health”[Major] OR “Physical Fitness/psychology”[Major] OR “Physical Fitness/physiology”[Major] OR “Health Status”[Major] OR “late effects”) AND adults.

### Study selection

Following the initial search, all identified citations will then be screened for duplications. First, titles and abstracts will be screened, and articles will be excluded if they do not meet basic inclusion criteria. Articles will be excluded if they address a population other than cancer or are not original articles (reviews, meta-analyses, case studies, case series, opinion pieces, editorials, study protocols, conference papers and efficacy/effectiveness trials will be excluded). For the second screening, articles of potentially eligible studies will be examined based on the age criteria. Only studies that address older patients with cancer (exclusively elderly ≥65 years, or mean/median age ≥ 70 years in age-mixed samples), compared older cancer patients with younger cancer patients or with older non-cancer controls will be included. The final full text screening of the remaining studies will be conducted independently by the first two authors (FS, AM). Articles will be excluded if no validated QoL assessment tool was used, or no results on QoL in older cancer patients are reported and thus no conclusions regarding the research questions can be drawn. Any disagreements regarding the inclusion of studies will be resolved through discussion.

### Data extraction

Data will be extracted and summarized using a data extraction sheet that was created by the first author and discussed within the research team to ensure that key information will be captured. The following data will be extracted and rated: (i) study characteristics: first author, year of publication, country of the study, study design, sample size; (ii) patient characteristics: mean age, range of age, tumor entity, tumor stage, time since diagnosis, type of cancer treatment, treatment stage, physical and mental comorbidities; (iii) QoL outcomes: QoL assessment tool, global QoL, physical functioning, mental/emotional functioning, social functioning, cognitive functioning, role functioning, unmet needs, values, preferences, functional health; (iv) associated factors with QoL: sociodemographic, medical, psychosocial, geriatric (socioenvironmental circumstances (social support, partnership, financial burden), activities of daily living, cognitive function, physical health, unmet needs).

Information on the methodological quality of the studies will not be extracted from the articles, as it is not the intention of this review to highlight methodological biases. Instead, our approach is to obtain and describe information on important aspects of QoL in older patients with cancer in order to generate hypotheses for future studies and aid the development of age-sensitive assessment tools for QoL.

The draft of the data extraction form (Supplementary Table S1) will be modified and revised as necessary during the data extraction of the included articles. Modifications will be described in detail in the scoping review. Data will be extracted by the first author and will be reviewed by the second author for accuracy.

### Data analysis and presentation

Descriptive statistics of the included studies and participants characteristics will be provided. QoL dimensions relevant in older patients with cancer will be summarized descriptively. Factors associated with QoL (medical, psychosocial, sociodemographic, geriatric) will be categorized in order to identify overarching topics and allow for the identification of at risk populations and healthcare settings. This may help to provide additional context and insight into specific subgroups of patients. The categorization process will be discussed within the research team. Subgroup analyses will be carried out across studies that compare older cancer survivors with younger as well as with older non-cancer cases.

## Discussion

Despite the increasing number of older adults with cancer, the knowledge regarding specific dimensions of QoL, life-related values and preferences in the elderly is still very sparse. The complexity of managing a cancer disease in elderly patients requires a deeper understanding of their healthcare aspects in order to improve tailored treatment, prevention programs and sustaining the patients’ functional health. This scoping review protocol describes the methodology, inclusion criteria, search strategy and study selection to assess QoL and associated factors in older adults with cancer.

There could be several limitations to this scoping review. First, the broad inclusion criteria with regard to medical characteristics, healthcare settings, as well as study types might limit the possibility to draw practical conclusions for patients in different healthcare settings. Different clinical settings can significantly impact the results related to QoL. However, we aim to give a broad overview of QoL in elderly cancer patients, which may serve as a basis for future studies focused on more specific elderly populations and healthcare settings. Additionally, we aim to investigate sociodemographic, medical, psychological and geriatric factors associated, that might help to draw conclusions regarding different patient populations. Second, we will not assess the methodological quality of the included studies, as the objective of this scoping review is not to highlight methodological biases, but to offer a broad perspective on QoL outcomes in older adults with cancer. Future systematic reviews may need to evaluate the methodological quality of included studies in greater detail. Finally, we will only include published studies in English language. Therefore, there might be a bias due to not capturing grey literature and culturally specific aspects of QoL, values and preferences might be difficult to emphasize. However, this decision was based on an initial search of relevant literature, which identifies a substantial number of studies to provide valuable data for this review.

This scoping review aims to provide valuable insights into central dimensions of QoL, including functional health, life-related values and preferences in older adults with cancer, and might thus guide future studies, interventions and healthcare planning.
