# Supplementary material for: Identifying central dimensions of quality of life including life-related values, preferences and functional health in older patients with cancer: a scoping review protocol
Source: Front Psychol. 2024 Oct 22;15:1455825. doi: 10.3389/fpsyg.2024.1455825 (PMC11534725; doi:10.3389/fpsyg.2024.1455825)
Supplement: Supplementary file 2 [file Table_2.DOCX]

**Supplementary Information**

**Table S1. Data extraction sheet draft – Scoping Review QoL in older patients with cancer**

| **#Number of Study** | **First Author** | **Title** | **Year of Publication** | **Country** | **Study design** | **Follow-Up period (longitudinal studies)** | **Sample Size** | **Comparison to…** |
| --- | --- | --- | --- | --- | --- | --- | --- | --- |
|  | Name of first author | Title of the study | Year | Where patients were recruited | cross-sectional, longitudinal, mixed methods | Months / Years | Cancer patients | … younger cancer patients (<65)  … elderly non-cancer cases |
| #1 |  |  |  |  |  |  |  |  |
| #2 |  |  |  |  |  |  |  |  |

| **#Number of Study** | **Age Mean/Median** | **Age Range** | **Age-mixed sample** | **Tumor Entities** | **Tumor Stage** | **Time Since Diagnosis** | **Comorbidities reported** | **Information on Comorbidities** |
| --- | --- | --- | --- | --- | --- | --- | --- | --- |
|  | Years | Years | Yes/No | List all included tumor entities | UICC, TNM, curative / palliative | Months/Years | Yes/No | Number of comorbidities, most frequent comorbidities etc. |
| #1 |  |  |  |  |  |  |  |  |
| #2 |  |  |  |  |  |  |  |  |

| **#Number of Study** | **QoL assessment tool** | **QoL global (results)** | | **QoL global (associated factors)** | | **QoL global (comparison group)** | | **Physical QoL (results)** | | **Physical QoL (associated factors)** | | **Physical QoL (comparison group)** | | **Mental QoL (results)** | | **Mental QoL (associated factors)** | | **Mental QoL (comparison group)** |  |
| --- | --- | --- | --- | --- | --- | --- | --- | --- | --- | --- | --- | --- | --- | --- | --- | --- | --- | --- | --- |
|  | Name of used validated tool | Briefly describe results | | List associated factors | | Younger cancer patients of elderly non-cancer cases | | Briefly describe results | | List associated factors | | Younger cancer patients of elderly non-cancer cases | | Briefly describe results | | List associated factors | | Younger cancer patients of elderly non-cancer cases |  |
| #1 |  |  | |  | |  | |  | |  | |  | |  | |  | |  |  |
| #2 |  |  | |  | |  | |  | |  | |  | |  | |  | |  |  |
| **#Number of Study** | **Symptom scales (results)** | | **Symptom scales (associated factors)** | | **Symptom scales (comparison group)** | | **Social functioning (results)** | | **Social functioning (associated factors)** | | **Social functioning (comparison group)** | | **Cognitive functioning (results)** | | **Cognitive functioning (associated factors)** | | **Cognitive functioning (comparison group)** | | |
|  | Briefly describe results | | List associated factors | | Younger cancer patients of elderly non-cancer cases | | Briefly describe results | | List associated factors | | Younger cancer patients of elderly non-cancer cases | | Briefly describe results | | List associated factors | | Younger cancer patients of elderly non-cancer cases | | |
| #1 |  | |  | |  | |  | |  | |  | |  | |  | |  | | |
| #2 |  | |  | |  | |  | |  | |  | |  | |  | |  | | |

| **#Number of Study** | **Role functioning (results)** | **Role functioning (associated factors)** | **Role functioning (comparison group)** | **Activities of daily living** | **Unmet Needs** | **Values, Preferences** | **Functional Health** | **Conclusion of the study** |
| --- | --- | --- | --- | --- | --- | --- | --- | --- |
|  | Briefly describe results | List associated factors | Younger cancer patients of elderly non-cancer cases | Briefly describe results | Conclusions regarding values and preferences | Conclusions regarding values and preferences | Conclusions regarding functional health | Briefly describe |
| #1 |  |  |  |  |  |  |  |  |
| #2 |  |  |  |  |  |  |  |  |
